# What is the correct level of claudication pain to prescribe? Universal inconsistency within guidelines, a painful issue

**DOI:** 10.1177/17085381231155940

**Published:** 2023-02-04

**Authors:** Sally A Seed, Amy E Harwood, Jonathan Sinclair, Anselm Egun, Stefan T Birkett

**Affiliations:** 1 School of Sport and Health Sciences, 6723 University of Central Lancashire, Preston, UK; 2 Centre for Sports, Exercise and Life Sciences, 120958 Coventry University, Coventry, UK; 3 6724 Lancashire Teaching Hospitals NHS Foundation Trust, Preston, UK; 4 Department of Sport and Exercise Sciences, Manchester Metropolitan University Institute of Sport, 5289 Manchester Metropolitan University, Manchester, UK

## Background

Peripheral artery disease (PAD) is an atherosclerotic cardiovascular condition affecting the lower limbs. A classic symptom of PAD is intermittent claudication (IC), which precipitates on exertion and is relieved with rest.^1^ Supervised exercise programmes (SEPs) are first line treatments for patients with IC.^2,3^ Despite the benefits of exercise, there are inconsistencies between guidelines regarding the recommended level of prescribed claudication pain. The aim of this commentary is to highlight the limitations of current guidance which will lead to variability in care.

The National Institute of Health and Care Excellence (NICE),^4^ American Heart Association^5^ and British Association of Sport and Exercise Sciences^6^ recommend exercising to maximal claudication pain. Supporting this, an early meta-analysis showed walking to near maximal pain to be most beneficial at improving maximal walking and pain-free walking distances. Indicating that greater amounts of ischaemia induced may produce greater haemodynamic and metabolic adaptations.^7^ Furthermore, high-intensity walking, eliciting moderate–severe ischaemic leg symptoms, was superior to low-intensity without ischaemic leg symptoms.^8^

Conversely, the American College of Sports Medicine^3^ and Exercise and Sports Science Australia^9^ suggest moderate pain is most beneficial. A systematic review suggested mild to moderate claudication pain yields optimal results in walking distance and cardiorespiratory fitness when compared to maximal pain.^10^ The conclusions were in line with the Vascular Disease Foundation and the American Association of Cardiovascular and Pulmonary Rehabilitation guidelines^11^ in that lower limb exercise should be performed to a threshold of mild–moderate pain.^12^

Despite the inconsistencies aforementioned, alarmingly several guidelines do not report a level of claudication pain to work towards,^10^ whilst others suggest working at a speed and gradient that induces claudication pain within 3–5 min, without specifying the intensity of the pain.^11^ As such, clinicians are not provided with clear guidance. A major issue is that guidelines are not fully inclusive of the evidence, as they do not consider pain-free exercise. A meta-analysis showed significant improvements in absolute and initial walking distance without inducing claudication pain.^13^ A recent systematic review also suggests pain-free SEPs elicit similar improvements in walking performance and functional outcomes compared to moderate pain.^14^

The missed consideration of pain-free SEPs and high claudication pain prescription may lead to poor uptake, as high claudication pain has been commonly cited as a barrier to exercise adherence and uptake of SEPs.^15,16^ Indeed patients taking part in a low pain SEP where 1.52 times more likely to complete the SEP than those in the high pain SEP.^16^ As SEPs are the first line of treatment for patients with PAD, guidelines must ensure greater participation occurs; consequently, patient ability and preference should be considered, so it is tolerable and beneficial for all patients.^17^

In conclusion, there are inconsistencies regarding the recommended level of claudication pain for clinicians to prescribe. Furthermore, there is no consideration for the evidence base regarding pain-free exercise. It is clear there is a need for a universal set of guidelines that consider optimising patient outcomes as well as uptake and adherence in relation to claudication pain. No research has directly compared maximal, pain-free and moderate claudication pain; however, trials are underway.^18^
